# Transcriptome Analysis Reveals the Algicidal Mechanism of *Brevibacillus laterosporus* against *Microcystis aeruginosa* through Multiple Metabolic Pathways

**DOI:** 10.3390/toxins14070492

**Published:** 2022-07-15

**Authors:** Yulei Zhang, Jieyi Li, Zhangxi Hu, Dong Chen, Feng Li, Xianghu Huang, Changling Li

**Affiliations:** 1 Department of Aquaculture, College of Fisheries, Guangdong Ocean University, Zhanjiang 524088, China; zhangyl@gdou.edu.cn (Y.Z.); lijieyi@stu.gdou.edu.cn (J.L.); huzx@gdou.edu.cn (Z.H.); chendong5609@163.com (D.C.); lifeng2318@gdou.edu.cn (F.L.); huangxh@gdou.edu.cn (X.H.); 2 Guangdong Laboratory of Marine Ecology Environment Monitoring and Warning, Zhanjiang 524088, China

**Keywords:** *Brevibacillus laterosporus*, *Microcystis aeruginosa*, transcriptome, algicidal effect, degradation, transporter, hydrolase

## Abstract

It is widely accepted that eutrophication has played an important role in the formation of harmful cyanobacterial blooms in recent decades, which impacts water quality and ecological environment and causes huge economic losses. Algicidal bacteria have a promising application prospect in controlling cyanobacterial blooms in aquaculture water. Here, the process of the algicidal bacterium *Brevibacillus laterosporus* strain Bl-zj acting on *Microcystis aeruginosa* was explored using transcriptome analysis to elucidate the algicidal mechanism. The results of the co-culture of bacterium and alga showed a strong alga-lysing effect of *B. laterosporus* against *M. aeruginosa* with an extreme morphology deformation of the algal cells. A total of 2744 differentially expressed genes of *B. laterosporus* were identified, which were mainly involved in the metabolism of amino acid, carbohydrate, and lipid. In the co-cultured group, the expression of genes mainly enriched in valine, leucine and isoleucine degradation, and fatty acid degradation were significantly increased. However, the expression of the genes related to ribosome were mainly inhibited. Transcriptome analysis showed that *B. laterosporus* obtained ATP and energy by the degradation of valine, leucine, isoleucine, and fatty acids, and destroyed algal cells by efflux pump transporters, secretion of hydrolytic enzymes, antibiotics, proteases, and other secondary metabolites, resulting in algal death and achieving the algicidal effect.

## 1. Introduction

In recent years, the outbreak of cyanobacterial blooms has caused severe water pollution and threatened the health of aquatic animals, resulting in significant economic losses for the aquaculture industry [1]. *Microcystis aeruginosa*, a common cyanobacteria species in aquatic ecosystems, can release microcystins, endangering the health and quality of cultured fish and shrimps. When a large number of *M. aeruginosa* accumulates in surface water, the transparency and dissolved oxygen levels of water decrease, which will further cause hypoxia and even the death of the aquatic animals [2]. Therefore, finding an effective method to control cyanobacteria blooms in aquatic ecosystems is crucial.

In comparison to physical and chemical methods for preventing and controlling cyanobacteria, biological methods have the advantage of being low cost and having high specificity and efficiency. Biological control has been recognized as an economic, effective, and ecologically harmless method to eliminate the harmful cyanobacteria [3]. Microorganisms, especially bacteria, play an important role in aquatic ecosystems [4]. As a group of bacteria that can inhibit and kill cyanobacteria, most algicidal bacteria lyse cyanobacteria indirectly by secreting extracellular substances or inhibit the grow through competing with them for limited nutrients. The specific or non-specific extracellular substances secreted by algae-lysing bacteria in the process of metabolism have an algal-lysing activity, which can destroy the structure of algal cells, causing them to disintegrate and die. In addition, some essential physiological functions of cyanobacteria can be destroyed and lead to the death at the later stage, including photosynthesis, reactive oxygen species (ROS) production, antioxidant system, and enzymatic activity [5]. Algicidal bacteria has significant prospects for water quality regulation as well as the prevention and control of cyanobacteria in aquaculture [6]. Lee et al. [7] isolated *Pseudoalteromonas* sp. strain A28 that can kill *Skeletonema costatum* strain NIES-324 by producing an extracellular serine protease. In vivo and in situ experiments conducted by Kim et al. [8] showed that the algae-lysing bacterium, *Xanthobacter autotrophicus* strain HYS0201-SM02, isolated from the surface water of a eutrophic lake displayed an algal-lytic activity against both cultured strain and natural colonial morphs of the *M. aeruginosa*. Yan et al. [9] screened *Streptomyces amritsarensis* HG-16, an actinomycete with high algicidal activity against *M. aeruginosa*, and found that *S. amritsarensis* could kill *M. aeruginosa* by secreting active substances, causing oxidative stress in algal cells, and strongly inhibiting microcystin synthesis of *M. aeruginosa*.

At present, research on the mechanism of algicidal bacteria against *M. aeruginosa* mainly focuses on the changes in cyanobacteria during the algae-lysis process. However, there are few studies on the changes in algicidal bacteria, particularly their transcriptome. Han et al. [10] investigated the gene expression and regulation of the algicidal fungus *Bjerkandera adusta* T1 using time-course transcriptomic analysis and speculated that the endopeptidase of polysaccharide lyases 8 (PL8) in this fungus might be the reason for the strong algicidal ability of *B. adusta* T1 and *Trametes versicolor* F21a. Dai et al. [11] analyzed the gene expression and regulation at time courses of *T. versicolor* F21a in the algae-killing process, which showed that the algae-killing mode of *T. versicolor* F21a might be related to enzyme decomposition and multiple metabolic pathways. Gao et al. applied proteomic analysis to investigate the algicidal process of *T. versicolor* F21a and found that some fungal enzymes may degrade lipopolysaccharides, peptidoglycans, and alginic acid of the algal cells [12]. Krachkovskii et al. [13] isolated a cyclic decapeptide cyclo peptide antibiotic loloatin A from the IGM52 strain of the Gram-positive spore-forming bacteria *B. laterosporus* that inhibits the growth of cyanobacteria.

*B. laterosporus* strain Bl-zj was isolated from intertidal soil and has been demonstrated to have algicidal activities against cyanobacteria [14,15,16]. In this work, the algicidal mechanism of the *B. laterosporus* strain Bl-zj against *M. aeruginosa* was investigated. By co-cultivating *B. laterosporus* and *M. aeruginosa* for 4 days, the changes in the algal morphology were observed. We also analyzed the differential gene expression of *B. laterosporus* during algal lysis and its role in the algicidal process from the transcriptome perspective. The algicidal mechanism of *B. laterosporus* was ascertained more comprehensively at the molecular level, providing a theoretical basis for water bloom control and a reference for the development of alternative microbial control agents for the prevention and control of cyanobacteria.

## 2. Results

### 2.1. Effect of B. laterosporus on the Cell Morphology of M. aeruginosa

**Figure 1:**
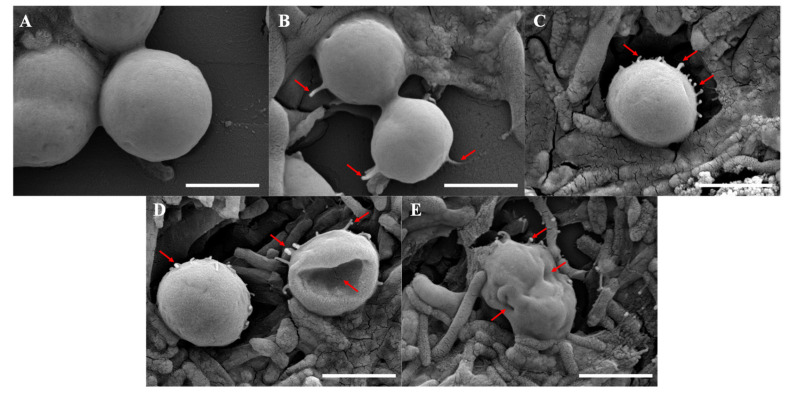
Morphology of *M. aeruginosa* (**A**), *M. aeruginosa* co-cultured with *B. laterosporus* for 1 d (**B**), 2 d (**C**), 3 d (**D**), and 4 d (**E**) by scanning electron microscopy (Scale bars = 2 μm). The red arrows showed the antenna-like mucus or cell depression and deformation.

Figure 1 showed the morphology of *M. aeruginosa*, as well as that of *M. aeruginosa* and *B. laterosporus* co-cultured for four days. The *M. aeruginosa* cells cultured solely had a regular morphology with smooth surface (Figure 1A). When *M. aeruginosa* was co-cultured with *B. laterosporus* for one day, there were still numerous algal cells and the bacteria adhered to the algal cells (Figure 1B). There were no significant morphological changes in the algal cells with a few antenna-like mucus presented (Figure 1B). On the second day, the surface of the algal cells had many antenna-like mucus and numerous bacteria gathered around (Figure 1C). However, the algal cells began to rupture or appeared deep depressions on the third day, making a gradually incomplete morphology (Figure 1D). Finally, the number of algal cells decreased sharply on the fourth day, and the cell surface shrank and deformed severely, with many mucus substances appearing on and around it (Figure 1E).

### 2.2. Transcriptome Sequencing, Assembly and Data Quality Analysis

Nine cDNA libraries were sequenced in three groups (Table 1). An average of 9,492,785,267 bp raw data and 63,285,235 raw reads were obtained from each sample. After filtering the adaptors and low-quality sequences from raw data, an average of 7,706,561,467 bp clean data and 51,377,076 clean reads were screened. After mapping to the reference genome, an average of 28,557,768 mapped reads were obtained. The base percentages of Q20 and Q30 in each sample were higher than 96.94% and 92.9%, respectively, indicating good assemble quality.

**Figure 2:**
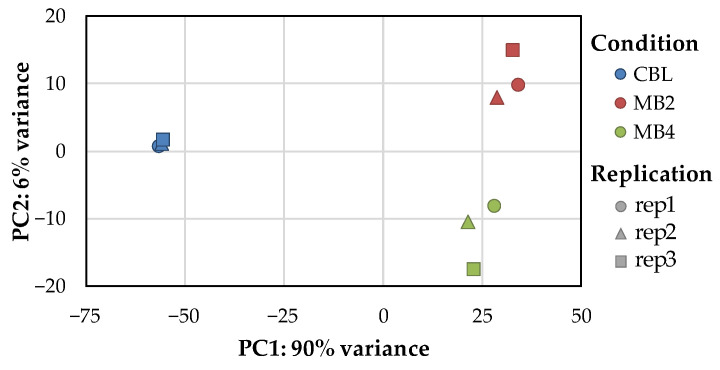
The principal component analysis (PCA) of each sample. The blue, red and green labels represent the treatment group of CBL, MB2 and MB4, respectively. The circle, triangle and square labels represent replication 1, replication 2 and replication 3 samples in each group, respectively.

Principal components analysis (PCA) was performed according to the expression of each sample to group similar samples. The closer the distance, the higher the similarity between the samples. As shown in Figure 2, the three biological replicates in each treatment group (CBL, MB2, MB4) were densely distributed, but different treatment groups were separated from others at a long distance, indicating good repeatability data.

### 2.3. Identification of Differentially Expressed Genes

**Figure 3:**
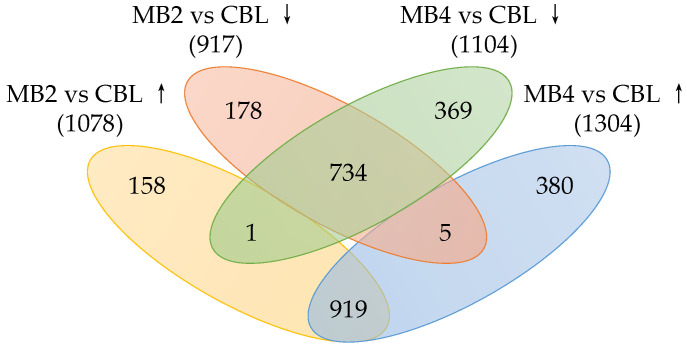
The number of differentially expressed genes (DEGs) at MB2 and MB4 groups compared with CBL group. “↑” and “↓” represent up- and down-regulated genes, respectively.

A total of 1995 differentially expressed genes (DEGs) were detected in MB2 vs. CBL, with 1078 being up-regulated and 917 being down-regulated (Figure 3). A total of 2408 DEGs were found in MB4 vs. CBL, with 1304 being up-regulated and 1104 being down-regulated (Figure 3). Both in MB2 vs. CBL and MB4 vs. CBL comparison, a total of 2744 DEGs were identified, of which 919 were up-regulated and 734 were down-regulated (Figure 3).

### 2.4. Enrichment Analysis of Differentially Expressed Gene Function

#### 2.4.1. Gene Ontology (GO) Pathway Analysis

**Figure 4:**
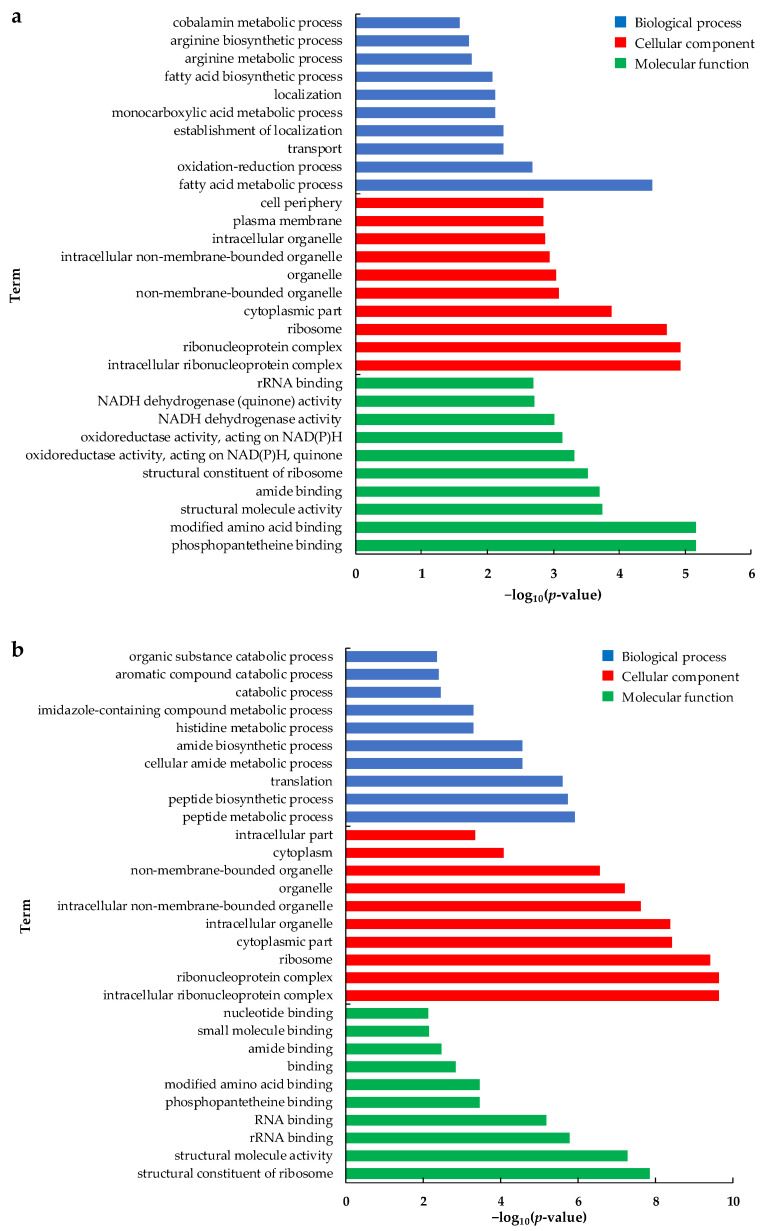
Gene ontology (GO) enrichment analysis of the differently expressed genes at two groups compared with CBL group. (**a**) MB2 vs. CBL comparison, (**b**) MB4 vs. CBL comparison.

GO pathway analysis results of the DEGs were classified according to biological processes (BP), cellular components (CC), and molecular functions (MF) (Figure 4). In MB2 vs. CBL, fatty acid metabolism process, oxidation-reduction process, and transport were significantly enriched in biological processes (Figure 4a). Intracellular ribonucleoprotein complex, ribonucleoprotein complex, ribosome, and cytoplasmic part were enriched in cell components. Phosphopantetheine binding, modified amino acid-binding, and structural molecule activity were all significantly enriched in molecular functions. Peptide metabolic process, peptide biosynthesis process, and translation were significantly enriched in biological processes in MB4 vs. CBL (Figure 4b). Intracellular ribonucleoprotein complex, ribonucleoprotein complex, ribosome, and cytoplasmic part were enriched in cell components. The structural constituent of ribosome, structural molecule activity, rRNA binding, and RNA binding was remarkably enriched in molecular functions.

#### 2.4.2. KEGG Pathway Analysis

**Figure 5:**
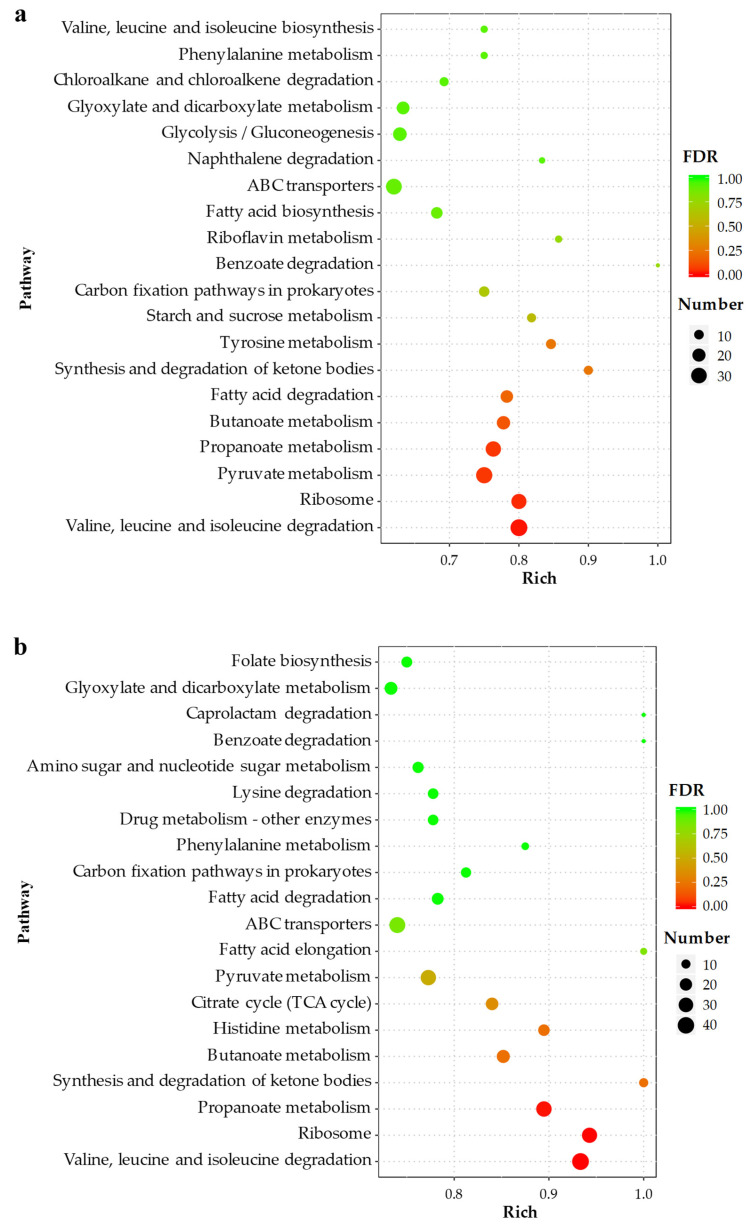
KEGG pathway enrichment analysis. (**a**) MB2 vs. CBL comparison, (**b**) MB4 vs. CBL comparison.

KEGG pathway analysis revealed that the DEGs were enriched in 113 pathways in MB2 vs. CBL and 114 pathways in MB4 vs. CBL. Table 2 shows that the two groups were mainly enriched in amino acid metabolism, carbohydrate metabolism, and lipid metabolism at level 2. The first 20 pathways with the most significant difference between the two groups are shown in Figure 5. Except for ribosomes being involved in genetic information processing, and ABC transporters being involved in environmental information processing, all the other pathways were associated with metabolism. The degradation of valine, leucine, and isoleucine were the pathways with the most significant difference between the two groups and had the largest number of DEGs. In addition, there were significant differences in ribosome and propanoate metabolism between the two groups, and more DEGs were enriched.

### 2.5. Analysis of Differentially Expressed Genes

#### 2.5.1. Degradation of Valine, Leucine, and Isoleucine and Fatty Acids

The enrichment analysis of differential genes showed that differential genes in the two groups were significantly enriched in the degradation pathways of valine, leucine, and isoleucine, as well as fatty acid degradation pathways, and the differential genes in the pathways were further analyzed (Table S1). In valine, leucine, and isoleucine degradation pathways, there were 36 DEGs in MB2 vs. CBL, with 27 DEGs up-regulated and 9 DEGs down-regulated. There were 42 DEGs in MB4 vs. CBL, in which 29 and 13 DEGs were up- and down-regulated, respectively. In the fatty acid degradation pathway, there were 18 DEGs in MB2 vs. CBL, with 14 and 4 DEGs being up- and down-regulated, respectively. There were 18 DEGs in MB4 vs. CBL, including 13 up-regulated and 5 down-regulated. Moreover, 36 genes related to valine, leucine, and isoleucine degradation and 17 genes related to fatty acid degradation were differentially expressed in both groups. In addition, 14 genes were involved in these two pathways (i.e., gene2408, gene0848, gene2681, gene4067, gene1093, gene2412, gene1125, gene1123, gene1908, gene0849, gene1122, gene1826, gene0117, and gene2964).

**Figure 6:**
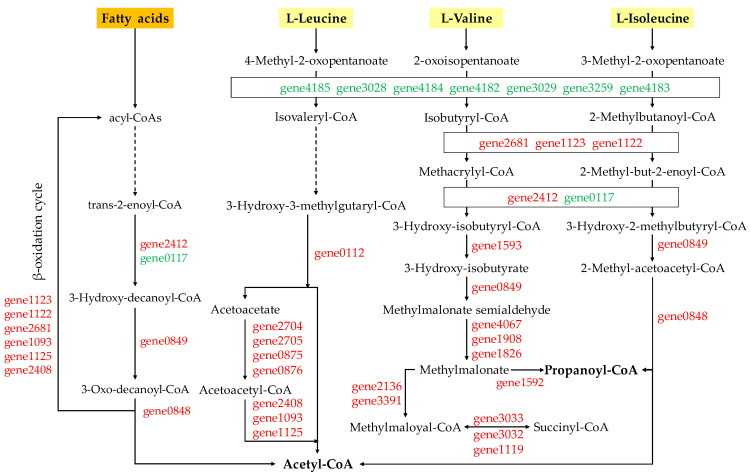
Pathways of valine, leucine, isoleucine and fatty acids degradation. (Red and green represent up-regulated and down-regulated genes, respectively.)

The degradation processes of valine, leucine, and isoleucine were mainly divided into three reactions (Figure 6). Transamination occurred in branched-chain amino acid, started with the removal of the amino group by aminotransferase or leucine dehydrogenase, giving alpha-keto acid. Then, oxidative decarboxylation was followed by the coupling dehydrogenation of the corresponding acyl-coenzyme A (acyl-CoA) derivative by a branch-ketoate dehydrogenase complex. Finally, the acyl-CoA derivatives of branched-chain amino acids were further converted to acetyl-CoA and propionyl-CoA through a separate branched-chain amino acid decomposition pathway. The expression of oxidative decarboxylation-related genes was down-regulated at 2nd day and 4th day but up-regulated in the subsequent generation of acetyl-CoA and propionyl-CoA. Most of the differential genes in fatty acid degradation pathway were also up-regulated.

#### 2.5.2. Transporter-Related Differential Genes

The efflux pump is an important transporter. Three families, namely ABC transporter, MFS, and RND, were identified in the genome of *B. laterosporus* strain Bl-zj. The transporters in the up-regulated genes were screened (Table 3). There were 28 transporter-related DEGs in MB2 vs. CBL and 24 transporter-related DEGs in MB4 vs. CBL, most of which were ABC transporter-related genes, with some MFS and RND genes up-regulated. The efflux transporter protein, MFP subunit (gene3336) was significantly up-regulated on 4th day, with log_2_(fold change) of 5.41. In addition, some transporter genes were up-regulated, such as oligopeptide transporter, OPT family (gene1164), cyclic peptide transporter family protein (gene3098), proton-coupled thiamine transporter *ThiT* (gene2438), inner membrane transporter *ycaM* (gene1184), etc.

#### 2.5.3. Hydrolase and Protease Related Differential Genes

Decomposition enzymes play an important role in the degradation of *M. aeruginosa* by *T. versicolor* F21a [11]. Hydrolases and proteases in the up-regulated genes were screened out in this work (Table 4). Cell wall hydrolase *CwlJ* (gene2308) exhibited ultra-high up-regulation in both groups, with log_2_(Fold Change) values on 2nd day and 4th day of 10.39 and 8.98, respectively. Furthermore, the expression levels of glycoside hydrolase, family 18 (gene3428) and glycosyl hydrolas (gene0800) were also up-regulated in the two groups. In addition, peptidase M23 (gene0479, gene1871), M23/37 (gene3430), M20 (gene0291) family proteins, minor extracellular protease *Vpr* domain (gene1081) and serine protease *HtrA* (gene0588) were also up-regulated in the two groups, with log_2_(Fold Change) values ranged from 3.48 to 8.58.

#### 2.5.4. Biosynthesis-Related Differential Genes of Other Secondary Metabolites

Genes in the biosynthesis pathway of other secondary metabolites were screened out from DEGs (Table 5). As can be seen, the genes related to novobiocin biosynthesis, prodigiosin biosynthesis, acarbose and validamycin biosynthesis, streptomycin biosynthesis, monobactam biosynthesis, carbapenem biosynthesis, and phenazine biosynthesis changed significantly. Among them, the genes related to prodigiosin biosynthesis, acarbose and validamycin biosynthesis, streptomycin biosynthesis, and monobactam biosynthesis were up-regulated, while the genes related to carbapenem biosynthesis were down-regulated.

### 2.6. Results of qRT-PCR

**Figure 7:**
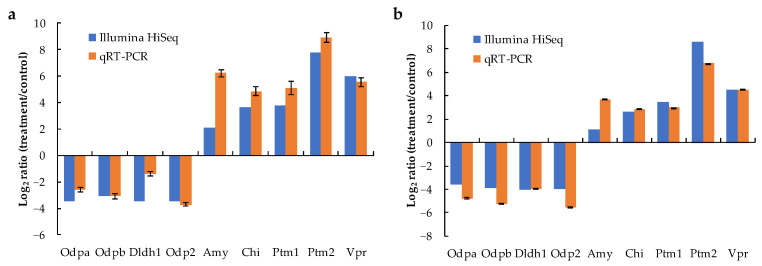
Comparison of the expressions of nine DEGs determined by Illumina HiSeq 2500 sequencing and qRT-PCR (The qRT-PCR data of up-regulated genes were redrawn from Zhang et al., 2021 [15]). (**a**) MB2 vs. CBL comparison, (**b**) MB4 vs. CBL comparison.

Nine genes were selected for validating RNA-seq results using qRT-PCR assay (Figure 7). Although the fold changes were not identical, the up- and down-regulation patterns of the nine genes presented the same trend in RNA-seq and qRT-PCR results, indicating that the transcriptome results were reliable.

## 3. Discussion

We found that metabolism and ribosome-related pathways through enrichment of GO and KEGG pathways was mainly involved in the algicidal effect of *B. laterosporus* strain Bl-zj against *M. aeruginosa*. The genes related to amino acid metabolism, carbohydrate metabolism, lipid metabolism, energy metabolism, biodegradation of harmful substances, and biosynthesis of other secondary metabolites were mainly up-regulated. The up-regulation of the first four pathways may indicate that *B. laterosporus* needs energy to secrete extracellular substances and grow. The up-regulation of genes in the latter two pathways may indicate that *B. laterosporus* secretes substances to inhibit cyanobacteria while resisting the possible harmful effects by *M. aeruginosa*. Morphology and ultrastructure of microbial cells were prone to modification or even destruction when exposed to toxic substances [17]. The cells of *M. aeruginosa* began gradually to rupture after one day of co-culture, which was largely affected by the toxic substances released by *B. laterosporus*.

Bacteria can degrade branched-chain amino acids (valine, leucine, and isoleucine) and fatty acids into small molecules and use them as carbon and energy sources to obtain ATP and energy. Differential genes are mainly involved in these two degradation pathways in the enrichment analysis of differential proteins showing that *T. versicolor* F21a decomposes *M. aeruginosa* [11]. Acetyl-CoA is an important cofactor in microbial cells and also an important intermediate metabolite of energy metabolism that plays a key role in the metabolic process. Acetyl-CoA can enter the tricarboxylic acid cycle and oxidative phosphorylation, releasing energy and producing ATP. The genes related to fatty acid degradation were mainly up-regulated on 2nd day and 4th day of co-culture, while the genes related to oxidative decarboxylation in the degradation of valine, leucine, and isoleucine were down-regulated, but the genes related to acetyl-CoA and propionyl-CoA were up-regulated in the subsequent generation. This indicates that the process of producing acetyl-CoA is not hindered, which may be due to the high content of chain acyl-CoA derivatives of branched-chain amino acids in cells, allowing them to avoid deamination and oxidative decarboxylation of branched-chain amino acids to ensure that the subsequent process acetyl-CoA production is normal. Acetaldehyde dehydrogenase (gene4067) can convert acetaldehyde into acetic acid, which is further converted into acetyl-CoA. On 2nd day and 4th day of co-cultivation, the log_2_(fold change) of gene4067 was 6.45 and 4.51, respectively. Acetyl-CoA content may be increased by overexpressing acetaldehyde dehydrogenase in *Saccharomyces cerevisiae* [18], which implies that the production of ATP may increase.

The efflux pump participates in a variety of physiological functions in bacteria and is crucial for the pathogenic process of bacteria. The ABC transporter family plays the most important and extensive role in the efflux pump system [19]. Environmental adaptability and bacterial viability are both dependent on ABC family transporters. The up-regulation of the expression of ABC transporters indicates an increase in the transport of substances inside and outside the cell. The transporters transfer the compounds extracellularly secreted by the bacteria during the process of Bl-zj algal degradation; on the other hand, the bacterium absorb the nutrients decomposed and released by the cyanobacteria after death, transporting and using them in the cells. Moreover, ABC transporters require ATP for material transport, which may also be the reason for strengthening the decomposition of branched-chain amino acids and fatty acids to produce acetyl-CoA and generate more energy. In a study on the algae-killing mode and stability of ZFX1 [20], it has been shown that the supernatant of ZFX1 could destroy the structure of the algal cell membrane, making the membrane system hard, and thus destroying the function of the membrane system. *B. laterosporuswas* has been shown to secrete bioactive metabolites that destroy the *Oscillatoria*’ system of membrane, photosynthesis, and antioxidant enzyme, which would deprive its normal physiological and metabolic function [14]. This observation also indicates that transporters play an important role in algae-lysing bacteria by releasing algae-lysing substances.

During the algae-lysing process of *Tramates versicolor* F21a, the activities of glycoside hydrolase, coenzyme, carbohydrate esterase, and polysaccharide lyases were significantly up-regulated, suggesting that these enzymes may degrade lipopolysaccharide, peptidoglycan, and alginate in algal cells [12]. In our work, the high up-regulated glycoside hydrolase family 18 (GH18) was chitinase, which is an antagonistic substance against pathogenic bacteria with an inhibitory effect on cyanobacteria. Hydrolases in algae-lysing bacteria can dissolve the cell wall of cyanobacteria and kill the algal cells. Hu et al. [21] found a β1,3-glucanase in the marine bacteria *Microbulbifer* sp. ALW1 can digest laminarin in the cell wall of brown algae. On the second day and fourth day, GH18 and cell wall hydrolase of *B. laterosporus* were significantly up-regulated. During the algae degradation, *B. laterosporus* releases hydrolases into the extracellular space, which may destroy the living algal cells or hydrolyze dead algal cells. Whatever function the hydrolases play, they are all important substances required by *B. laterosporus* in the process of algal degradation. Moreover, *B. laterosporus* secretes hydrolase extracellularly, which also requires the use of efflux pumps to facilitate the flow of material.

Antibiotics are secondary metabolites produced during bacterial growth that have an anti-pathogen effect and further activities, which can interfere with the development of other live cells. The expression of some genes related to antibiotic biosynthesis in Bl-zj was up-regulated during the algal degradation, suggesting that the released antibiotics may increase during this process [22]. Many antibiotics are cytotoxic, and the up-regulated antibiotics in these biosynthetic pathways are potential algae-lysing substances of *B. laterosporus*. Among them, the expression of prodigiosin biosynthesis pathway is up-regulated. Prodigiosin (PG) is a kind of natural pigment with insecticidal activity [23], bactericidal [24], and algicidal [25] effects. In addition to inhibiting *Heterosigma akashiwo* [26] and *Phaeocystis globosa* [27], PG isolated from *Hahella* sp. KA22 displays excellent algacidal action against *M. aeruginosa* [28]. PG causes the production of reactive oxygen species (ROS) by *M. aeruginosa* and lipid peroxidation of the algal cells, as well as destroys the function of the membrane system and the light system of the algal cells, resulting in the death of *M. aeruginosa*. *Serratia marcescens* LTH-2 secreted PG of strong degradation activity on *M. aeruginosa* strains TH1, TH2, and FACHB 905 [29]. In addition, the biosynthesis-related genes of acarbose and validamycin, streptomycin, monobactam, and novobiocin were also up-regulated. Streptomycin is toxic to *M. aeruginosa* and *Chlorella vulgaris*, affecting the transcription of photosynthesis-related genes in these two algae and preventing electron transport and excessive production of ROS [30]. Novobiocin induces DNA damage and apoptosis in CBL cells by activating ROS production [31].

In addition to hydrolytic enzymes and antibiotics, extracellular proteases may be involved in the degradation of cyanobacteria. M23 family peptidase and *Vpr* were up-regulated on 2nd day and 4th day. The M23 family includes amide enzymes or endopeptidases that can degrade bacterial cell walls. These peptidases can specifically cleave the link between N-acetylmuramoyl-L-alanine amidase and peptide bridges in the bacterial cell wall peptidase network. Bacteria secrete M23 family proteases to degrade the cell wall of other bacteria in order to perform defensive and offensive functions. The cell wall of *M. aeruginosa* is also composed of peptidoglycan. M23 family proteins may destroy the cell wall of *M. aeruginosa* [32]. *Vpr* is an extracellular alkaline serine protease. Bhaskar et al. reported that *Bacillus proteolyticus* CFR3001 can secrete an alkaline protease to degrade the cell wall of pathogenic bacteria and lyse the cells [33]. According to the results of Du et al. [34] extracellular proteases released by *T. versicolor* F21a may be the key to *Microcystis* degradation, whose rate was positively correlated with the activities of these enzymes. Zeng et al. [35] found that *P. chrysosporium* could degrade soluble proteins in algal cells, block nutritional supply, and effectively inhibit algal growth. Barbieri et al. [36] studied a transcription regulatory factor *CodY* in low-G+C Gram-positive bacteria and reported that *Vpr* became one of the most abundant proteins in the exoproteome of *Bacillus subtilis* in the *CodY*-null mutant strain.

Competition or synergy often exits between organisms in a common environment [37]. Previous study analyzed the transcriptional changes of *M. aeruginosa* co-cultured with *B. laterosporus* [16], which suggested that *B. laterosporus* could block the electron transport by attacking the PSI system and complex I of *M. aeruginosa*, affecting the energy acquisition and causing oxidative damage. This further led to the lipid peroxidation of the microalgal cell membrane, resulting in algal death. In this study, under the stimulation of *M. aeruginosa*, *B. laterosporus* could inhibit the growth of cyanobacteria and even destroy its cells by regulating the transcription levels. By increasing the secretion of functional substances such as antibiotics, hydrolases and proteases, *B. laterosporus* would cope with the competition brought by cyanobacteria in the living environment. In addition, as protein synthesis/turnover in ribosomes is an energy-intensive cellular process, the decline in ribosomal biogenesis may serve as an alternative mechanism to achieve energy-saving and healthy aging [38]. There were significantly enriched in ribosome pathway between the two groups, and most genes related in nucleotide metabolism and translation were down-regulated. Therefore, it is speculated that *B. laterosporus* may delay its life cycle to improve the survival ability and competitiveness by saving energy, to take an advantage in niche competition.

## 4. Conclusions

The mechanism of algal lysis of *B. laterosporus* against *M. aeruginosa* was investigated in this study based on the analysis of morphology and transcription level. *B. laterosporus* inhibits the growth of *M. aeruginosa*, causing serious damage to its cell morphology. GO enrichment analysis showed that the group MB2 vs. CBL exhibited the largest enrichment in intracellular ribonucleoprotein complex, ribonucleoprotein complex, phosphopantetheine binding, and fatty acid metabolic process. MB4 vs. CBL was the most enriched in intracellular ribonucleoprotein complex, ribonucleoprotein complex, structural constituent of ribosome, and peptide metabolic process. In the KEGG pathway, the two groups were mainly enriched in amino acid metabolism, carbohydrate metabolism, lipid metabolism, and energy metabolism. Transcriptome analysis showed that *B. laterosporus* produced ATP and energy by degrading valine, leucine, isoleucine, and fatty acids, and destroyed the algal cells using efflux pump transporters, secretion of hydrolytic enzymes, antibiotics, proteases, and other secondary metabolites such as prodigiosin, resulting in cyanobacteria death and achieving the effect of algae lysis.

## 5. Materials and Methods

### 5.1. Co-Cultivation of B. laterosporus and M. aeruginosa

*M. aeruginosa* FACHB 905 was purchased from the Freshwater Algae Culture Collection at the Institute of Hydrobiology, Wuhan, China. It was cultured on BG11 media [39] at 28 ± 1 °C under 50 µmol·m^−2^·s^−1^, and a light–dark cycle of 12 h: 12 h for seven days to reach the exponential growth stage. *B. laterosporus* strain Bl-zj was isolated from intertidal soil, activated and expanded in beef extract peptone liquid medium, then cultured at 30 °C and shaken at 150 rpm for 12–18 h to reach the exponential growth stage.

The cell concentrations of *M. aeruginosa* and *B. laterosporus* Bl-zj were adjusted to 1 × 10^7^ cells·mL^−1^. A total of 50 mL of *M. aeruginosa* and 50 mL of the bacterium (cell density ratio = 1:1) were added to 400 mL of the BG11 medium. The control group (CBL) consisted of 50 mL of *B. laterosporus* added to 450 mL of the BG11 medium. Three parallel experiments were set up in each group. The treatments were placed in a 28 °C incubator, under 50 µmol·m^−2^·s^−1^, and a light–dark cycle of 12 h: 12 h for static culture, with three times manually shaken a day. The experiment lasted 4 days when the co-cultured group was turned yellow and the cyanobacteria biomass was at a low proportion.

### 5.2. Specimen Preparation for Scanning Electron Microscopy

A total of 20 mL of the samples were obtained from each group every other day, and then the supernatant was removed after centrifugation. After three times of rinse, 1 mL of 2.5% glutaraldehyde solution was added for fixation. Gradient dehydration was subsequently performed by a series of ethanol solutions (30, 50, 70, and 80%). The specimens were centrifuged to remove the supernatant after 15 min. After two washes with 100% ethanol, 500 μL of 100% ethanol was added to resuspend. Droplets of 10 μL of the resuspended sample were deposited on the surface of the microscope slide and frozen at −80 °C for 2 h. Using a freeze dryer (CryoStar NX50 HOVPD, Thermo Fisher Scientific, Waltham, MA, USA), the droplets were dried and fixed on the slide for observation using a scanning electron microscope (Tescan MIRA, 3XMH, Brno, s.r.o, Czech Republic).

### 5.3. Transcriptome Sample Processing and Sequencing

The cell concentrations of *M. aeruginosa* and *B. laterosporus* were adjusted to 1 × 10^7^ cells/mL and co-cultured at a ratio of 1:1. A single culture of *B. laterosporus* was set as the control group (CBL group). Bacterial specimens were collected from the culture medium on second day (MB2 group) and fourth day (MB4 group) of the experiment, immediately frozen using liquid nitrogen, and stored in a refrigerator at −80 °C. Three parallels were set for each group.

Total RNA was extracted from the samples using RNAprep Pure Plant Plus Kit (Polysaccharides & Polyphenolics-rich, TANGEN, Beijing, China). Following RNA concentration, purity and integrity were detected, rRNA in total RNA was removed, and the rRNA product was interrupted to synthesize double-stranded cDNA and complete cDNA repair. The product joints were connected, the connector was purified, the enriched fragment library was amplified by PCR, and the sequencing library was constructed. The high-throughput sequencing part of this experiment was conducted by Shanghai Parsono Biotechnology Co., Ltd. (Shanghai, China) using Illumina HiSeq 2500 platform for transcriptome sequencing.

### 5.4. Analysis of Differential Expressed Genes

The high-quality data filtered from original data with reference genome was compared and annotated. HTSeq 0.6.1p2 (http://www.huber.embl.de/users/anders/HTSeq, accessed on 30 October 2019) was used to count the number of reads on each gene as the original gene expression, and FPKM (Fragments Per Kilobase of exon model per Million mapped fragments) was used to standardize the expression. The fold change between the experimental group and the control group was calculated, and the conditions for screening differentially expressed genes were as follows: log_2_| Fold Change | > 1, significant *p* < 0.05, the number of unique differential genes between each comparison group was counted according to the different analysis results. Principal components analysis was executed with PCAtools in R language (vision 4.0.0).

GO (http://geneontology.org/, accessed on 30 October 2019) and KEGG databases (https://www.kegg.jp/, accessed on 30 October 2019) were used to annotate the differential genes. The gene groups that potentially have an algicidal effect were screened for analysis.

### 5.5. Quantitative PCR Verification

The results of the mRNA-seq analysis were verified by fluorescence quantitative PCR (qPCR). RNA was reverse-transcribed into cDNA using HiScriptR III RT SuperMix for qPCR (+gDNA wiper) (R323-01, Vazyme Biotech, Nanjing, China). qPCR was performed using ChamQ Universal SYBR qPCR Master Mix (Q711-02/03, Vazyme Biotech, Nanjing, China). The primers were designed according to the whole genome sequence of Bl-zj with 16S rRNA gene used as the reference gene and were synthesized by Jinweizhi Biotechnology Co., Ltd. (Suzhou, China) (Table S2).

## Figures and Tables

**Table 1:** Overview of *B. laterosporus* transcriptome sequencing data.

Sample	Raw Data (bp)	Raw Reads No.	Clean Data (bp)	Clean Reads No.	Mapped Reads No.	Q20 (%)	Q30 (%)
CBL1	8,378,192,100	55,854,614	7,357,141,200	49,047,608	48,325,154	98.14	94.54
CBL2	8,746,394,400	58,309,296	7,617,902,100	50,786,014	49,954,121	98.07	94.42
CBL3	7,970,677,500	53,137,850	7,125,755,100	47,505,034	46,792,179	98.05	94.30
MB2_1	11,260,710,900	75,071,406	9,110,216,700	60,734,778	15,744,400	97.73	94.22
MB2_2	9,961,599,300	66,410,662	7,954,650,900	53,031,006	12,159,967	97.62	93.88
MB2_3	9,498,113,100	63,320,754	7,660,962,000	51,073,080	12,936,077	97.65	93.90
MB4_1	10,501,108,500	70,007,390	8,108,475,900	54,056,506	15,140,888	97.72	94.15
MB4_2	9,475,501,500	63,170,010	7,402,896,600	49,352,644	42,864,396	97.29	93.41
MB4_3	9,642,770,100	64,285,134	7,021,052,700	46,807,018	13,102,733	96.94	92.90
Average	9,492,785,267	63,285,235	7,706,561,467	51,377,076	28,557,768	97.69	93.97

**Table 2:** Distribution of up- and down-regulated genes in KEGG pathways.

Level 2	MB2 vs. CBL			MB4 vs. CBL		
	Up	Down	Total	Up	Down	Total
Amino acid metabolism	122	47	169	131	76	207
Carbohydrate metabolism	93	90	183	93	116	209
Lipid metabolism	51	15	66	52	22	74
Metabolism of cofactors and vitamins	28	36	64	26	41	67
Energy metabolism	34	16	40	32	21	53
Nucleotide metabolism	6	32	38	7	43	50
Translation	0	33	33	1	47	48
Xenobiotics biodegradation and metabolism	24	14	38	24	21	45
Membrane transport	13	21	34	13	28	41
Metabolism of other amino acids	18	9	27	13	16	29
Replication and repair	1	9	10	7	14	21
Folding, sorting and degradation	4	10	14	6	13	19
Biosynthesis of other secondary metabolites	11	5	16	10	7	17
Metabolism of terpenoids and polyketides	11	4	15	11	6	17
Cellular community—prokaryotes	4	5	9	6	6	12
Infectious diseases: Bacterial	1	3	4	2	2	4
Signal transduction	3	0	3	3	1	4
Immune system	1	1	2	1	1	2
Transcription	0	1	1	0	2	2
Environmental adaptation	1	0	1	1	0	1
Cell growth and death	0	0	0	0	3	3
Glycan biosynthesis and metabolism	1	0	1	0	0	0

**Table 3:** DEGs related to transporters.

Gene ID	MB2 vs. CBL		MB4 vs. CBL		Description
	log_2_ (Fold Change)	*p*-Value	log_2_ (Fold Change)	*p*-Value	
gene2217	7.04	1.04 × 10^−^^17^	2.29	1.80 × 10^−^^11^	ABC transporter
gene3791	3.08	1.12 × 10^−^^3^	2.09	1.31 × 10^−^^3^	ABC transporter family protein
gene2788	3.56	2.60 × 10^−^^5^	1.68	1.13 × 10^−^^8^	ABC transporter family protein
gene4041	7.42	1.34 × 10^−^^8^	1.96	1.33 × 10^−^^2^	ABC transporter permease protein
gene2787	3.40	7.31 × 10^−^^6^	1.63	2.79 × 10^−^^2^	ABC transporter permease protein
gene0214	3.53	1.37 × 10^−^^5^	5.08	6.01 × 10^−^^14^	ABC transporter permease protein
gene3837	4.19	2.00 × 10^−^^15^	1.48	1.64 × 10^−^^3^	ABC transporter ATP-binding/permease protein *TycE*
gene3838	4.42	1.73 × 10^−^^7^	2.33	2.00 × 10^−^^3^	ABC transporter ATP binding/permease protein *TycD*
gene3493	2.68	7.63 × 10^−^^6^	6.01	2.16 × 10^−^^4^	ABC transporter substrate-binding protein
gene2004	3.42	2.74 × 10^−^^7^	3.01	1.19 × 10^−^^20^	ABC transporter ATP-binding protein
gene1946	2.47	5.55 × 10^−^^6^	4.62	3.49 × 10^−^^23^	ABC-2 type transporter family protein
gene1947	2.15	5.42 × 10^−^^6^	3.01	3.80 × 10^−^^22^	Putative ABC transporter ATP-binding protein
gene0517	6.50	9.88 × 10^−^^9^	1.12	1.45 × 10^−^^2^	Oligopeptide/dipeptide ABC transporter, ATP-binding, C-terminal domain protein
gene0714	2.15	1.09 × 10^−^^2^	1.45	1.66 × 10^−^^2^	Peptide permease, major facilitator family transporter
gene1164	7.78	2.54 × 10^−^^41^	5.61	1.46 × 10^−^^57^	Oligopeptide transporter, OPT family
gene2954	3.43	4.50 × 10^−^^3^	2.82	1.82 × 10^−^^6^	Putative bacteriocin export ABC transporter, lactococcin group
gene3098	3.31	1.67 × 10^−^^10^	1.85	4.72 × 10^−^^6^	Cyclic peptide transporter family protein
gene1210	1.83	3.92 × 10^−^^3^	4.71	5.95 × 10^−^^43^	Uncharacterized MFS-type transporter *YdgK*
gene1223	3.71	3.11 × 10^−^^8^	3.23	1.02 × 10^−^^24^	Glycerol-3-phosphate transporter
gene1160	1.33	5.63 × 10^−^^3^	–	–	Putative multidrug resistance ABC transporter ATP-binding/permease protein *YheH*
gene1161	1.60	1.44 × 10^−^^2^	–	–	Putative multidrug resistance ABC transporter ATP-binding/permease protein *YheI*
gene1174	1.12	4.87 × 10^−^^2^	–	–	ABC transporter family protein
gene1714	2.70	4.84 × 10^−^^2^	–	–	Phosphate ABC transporter, permease protein *PstA*
gene0216	3.44	2.08 × 10^−^^2^	–	–	ABC transporter substrate binding protein
gene3053	1.05	2.63 × 10^−^^2^	–	–	Oligopeptide ABC transporter permease protein
gene3055	1.74	2.24 × 10^−^^2^	–	–	Oligopeptide ABC transporter ATP binding protein
gene3056	1.39	3.32 × 10^−^^2^	–	–	Oligopeptide ABC transporter ATP binding protein
gene0492	1.89	1.72 × 10^−^^3^	–	–	Citrate transporter family protein
gene1184	–	–	6.92	2.76 × 10^−^^44^	Inner membrane transporter *ycaM*
gene2438	–	–	5.09	9.13 × 10^−^^12^	Proton-coupled thiamine transporter *ThiT*
gene2761	–	–	1.85	4.33 × 10^−^^10^	Formate/nitrite transporter
gene3336	–	–	5.41	1.44 × 10^−^^5^	Efflux transporter, RND family, MFP subunit
gene4037	–	–	1.41	1.03 × 10^−^^2^	ABC transporter family protein

**Table 4:** DEGs related to hydrolase.

Gene ID	MB2 vs. CBL		MB4 vs. CBL		Description
	log_2_ (FoldChange)	*p*-Value	log_2_ (FoldChange)	*p*-Value	
gene2281	1.47	4.43 × 10^−^^3^	1.54	1.24 × 10^−^^6^	Cof-like hydrolase family protein
gene2308	10.39	1.83 × 10^−^^9^	8.98	6.29 × 10^−^^27^	Cell wall hydrolase *CwlJ*
gene2656	2.72	7.69 × 10^−^^5^	3.27	2.63 × 10^−^^13^	Dienelactone hydrolase family protein
gene2741	1.97	8.71 × 10^−^^4^	1.18	6.71 × 10^−^^3^	Putative hydrolase
gene2775	1.30	2.05 × 10^−^^2^	1.87	3.04 × 10^−^^7^	HAD-superfamily hydrolase *YhcW*
gene3428	5.31	5.85 × 10^−^^5^	4.89	7.79 × 10^−^^8^	Glycoside hydrolase, family 18
gene3564	2.65	4.09 × 10^−^^4^	4.51	1.49 × 10^−^^34^	Putative polyketide biosynthesis zinc-dependent hydrolase *PksB*
gene3616	2.37	4.74 × 10^−^^4^	1.71	1.30 × 10^−^^4^	Alpha/beta hydrolase fold family protein
gene3797	4.40	8.05 × 10^−^^12^	3.60	2.13 × 10^−^^12^	Amidohydrolase family protein
gene0510	4.40	9.63 × 10^−^^17^	4.25	3.45 × 10^−^^33^	Fumarylacetoacetate (FAA) hydrolase family protein
gene0800	4.04	1.44 × 10^−^^2^	6.09	2.09 × 10^−^^4^	Glycosyl hydrolase
gene1702	2.73	9.47 × 10^−^^6^	4.61	2.82 × 10^−^^25^	Metal dependent phosphohydrolase
gene1917	3.10	3.78 × 10^−^^5^	5.27	7.59 × 10^−^^48^	Membrane-bound metal-dependent hydrolase
gene2086	1.21	3.56 × 10^−^^2^	1.25	3.42 × 10^−^^4^	Alpha/beta hydrolase fold family protein
gene2140	2.10	3.17 × 10^−^^2^	1.09	2.07 × 10^−^^3^	Amylopullulanase
gene3978	3.62	6.60 × 10^−^^4^	2.59	5.05 × 10^−^^16^	Chitinase A1
gene0479	3.74	1.88 × 10^−^^8^	3.48	1.75 × 10^−^^5^	Peptidase M23 family protein
gene1871	7.77	1.15 × 10^−^^17^	8.58	5.53 × 10^−^^18^	Peptidase M23 family protein
gene3430	5.06	9.12 × 10^−^^7^	3.75	1.63 × 10^−^^7^	Peptidase M23/M37 family protein
gene0291	5.00	2.48 × 10^−^^5^	3.73	7.53 × 10^−^^6^	Peptidase M20 family protein
gene1081	5.98	8.93 × 10^−^^17^	4.50	2.08 × 10^−^^3^	Minor extracellular protease *Vpr* domain protein
gene2099	2.44	1.79 × 10^−^^6^	2.32	2.70 × 10^−^^12^	Intracellular protease
gene0588	7.01	5.70 × 10^−^^5^	5.26	2.53 × 10^−^^17^	Serine protease *HtrA*
gene2096	4.37	7.44 × 10^−^^3^	–	–	Alpha/beta hydrolase fold family protein
gene1396	1.12	3.49 × 10^−^^2^	–	–	Peptidyl-tRNA hydrolase
gene2293	2.27	3.88 × 10^−^^3^	–	–	Oligoendopeptidase F
gene2996	1.52	1.58 × 10^−^^2^	–	–	Peptidase SpoIVB
gene2763	1.60	7.96 × 10^−^^4^	–	–	Metalloprotease YpwA
gene1264	–	–	4.10	1.38 × 10^−^^2^	Acetyltransferases and hydrolases with the alpha/beta hydrolase fold
gene1950	–	–	1.70	9.00 × 10^−^^5^	*MazG* nucleotide pyrophosphohydrolase domain protein
gene1974	–	–	1.63	9.93 × 10^−^^4^	Glycoside hydrolase family 18

**Table 5:** DEGs related to biosynthesis of other secondary metabolites.

Gene ID	MB2 vs. CBL		MB4 vs. CBL		Description
	log_2_(Fold Change)	*p*-Value	log_2_(Fold Change)	*p*-Value	
Novobiocin biosynthesis					
gene3280	2.01	1.82 × 10^−^^2^	1.18	1.39 × 10^−^^3^	Histidinol-phosphate aminotransferase
gene3516	−4.17	7.39 × 10^−^^18^	−2.84	9.13 × 10^−^^17^	Threonine-phosphate decarboxylase
Prodigiosin biosynthesis					
gene3924	4.36	6.65 × 10^−^^7^	3.83	1.70 × 10^−^^6^	Short chain dehydrogenase family protein
gene1867	5.08	9.86 × 10^−^^14^	4.62	2.10 × 10^−^^30^	3-oxoacyl-[acyl-carrier-protein] reductase
gene3734	6.01	4.80 × 10^−^^11^	4.52	7.65 × 10^−^^20^	Malonyl CoA-acyl carrier protein transacylase
gene3394	6.55	2.29 × 10^−^^33^	5.96	6.87 × 10^−^^66^	Malonyl CoA-acyl carrier protein transacylase
gene4412	1.37	6.10 × 10^−^^3^	–	–	Putative 3-oxoacyl-[acyl-carrier protein] reductase
gene4621	−2.01	8.40 × 10^−^^6^	−3.57	8.84 × 10^−^^25^	3-oxoacyl-[acyl-carrier-protein] reductase
gene4622	−2.30	3.87 × 10^−^^7^	−3.55	5.31 × 10^−^^25^	Malonyl CoA-acyl carrier protein transacylase
gene2031	–	–	−2.14	2.97 × 10^−^^10^	Enoyl-[acyl-carrier-protein] reductase
gene3922	–	–	−1.85	4.54 × 10^−^^5^	Malonyl CoA-acyl carrier protein transacylase
Acarbose and validamycin biosynthesis					
gene1620	3.30	8.16 × 10^−^^3^	2.25	4.18 × 10^−^^4^	CDP-glucose 4,6-dehydratase
Streptomycin biosynthesis					
gene1620	3.30	8.16 × 10^−^^3^	2.25	4.18 × 10^−^^4^	CDP-glucose 4,6-dehydratase
gene3643	2.42	4.89 × 10^−^^5^	–	–	Myo-inositol-1-phosphate synthase family protein
gene0637	–	–	1.81	9.29 × 10^−^^7^	Inositol monophosphatase family protein
Monobactam biosynthesis					
gene2420	1.97	1.33 × 10^−^^3^	1.75	1.95 × 10^−^^5^	Aspartokinase 2
gene2342	2.41	1.91 × 10^−^^5^	–	–	Sulfate adenylyltransferase
gene3298	−1.43	4.14 × 10^−^^3^	–	–	Dihydrodipicolinate reductase
Carbapenem biosynthesis					
gene0853	−1.04	3.28 × 10^−^^2^	−1.09	7.42 × 10^−^^3^	Glutamate-5-semialdehyde dehydrogenase
gene0853	–	–	−1.75	1.93 × 10^−^^7^	Glutamate-5-semialdehyde dehydrogenase
Phenazine biosynthesis					
gene3274	–	–	5.34	7.17 × 10^−^^9^	Anthranilate synthase component

## Data Availability

All data are presented in the manuscript or Supplementary Tables, no data uploaded elsewhere.

## Supplementary Materials

The following supporting information can be downloaded at: https://www.mdpi.com/article/10.3390/toxins14070492/s1, Table S1: DEGs related to valine, leucine and isoleucine degradation and fatty acid degradation.; Table S2: qPCR primer pairs used in this study.
